# VDR polymorphisms and their influence on oral squamous cell carcinoma susceptibility: a systematic review

**DOI:** 10.3389/froh.2026.1687857

**Published:** 2026-02-23

**Authors:** Mithra N. Hegde, Nireeksha, Harshitha Somanatha, Nishmitha N. Hegde, Manikandan Ravinanthanan, Usha Balan

**Affiliations:** 1 Nitte (Deemed to be University), AB Shetty Memorial Institute of Dental Sciences (ABSMIDS), Department of Conservative Dentistry and Endodontics, Mangaluru, India; 2 Nitte (Deemed to be University), AB Shetty Memorial Institute of Dental Sciences (ABSMIDS), Research Lab, Mangaluru, India; 3 Department of Restorative Dental Sciences, Faculty of Dentistry, Al Baha University, Al Bahah, Saudi Arabia; 4 Department of Oral Pathology and Microbiology, KMCT Dental College, Kozhikode, India

**Keywords:** *FokI*, oral squamous cell carcinoma, SNP, *TaqI*, VDR polymorphism

## Abstract

**Background:**

Oral Squamous Cell Carcinoma (OSCC) arises through an intricate interplay of underlying genetic traits and surrounding environmental conditions. Variations in the vitamin D receptor (VDR) gene, particularly single-nucleotide polymorphisms (SNPs), may contribute to individual susceptibility and disease progression.

**Objective:**

To conduct a systematic review evaluating the role of VDR gene polymorphisms in influencing susceptibility to OSCC.

**Methods:**

A systematic literature search was performed in PubMed, Scopus, and Google Scholar to identify human case-control studies evaluating VDR SNPs in relation to OSCC. The methodological quality and potential bias of the selected studies were assessed using the Newcastle-Ottawa Scale (NOS). Due to substantial heterogeneity among the studies in terms of population characteristics, genotyping methods, and outcome measures, a meta-analysis was not performed, and the findings were summarized descriptively.

**Results:**

From an initial pool of 90 records, five case-control studies met the inclusion criteria. Qualitative synthesis revealed consistent associations between polymorphisms in the VDR gene, particularly *Fok1* (rs2228570), *TaqI* (rs731236*)*, and *ApaI* (rs7975232), and in the *CYP24A1* (rs2296241) gene, which encodes a VDR-regulated enzyme involved in the catabolism of active vitamin D metabolites. These variants have also been explored for their potential association with oral potentially malignant disorders (OPMDs), including leukoplakia, oral submucous fibrosis, and oral erythroplakia, which may undergo malignant transformation to OSCC. Most of the included studies demonstrated a low risk of bias and supported the role of these SNPs in OSCC susceptibility, as well as their potential prognostic relevance.

**Conclusion:**

VDR polymorphisms may contribute to OSCC risk by affecting immune and cellular pathways. Although findings are promising, larger, multi-ethnic studies are needed to confirm their clinical significance.

**Systematic Review Registration:**

https://www.crd.york.ac.uk/PROSPERO/view/CRD420251105619, PROSPERO CRD420251105619.

## Introduction

Cancers of the oral cavity are among the most widespread malignancies of the head and neck, with OSCC being the predominant histological type. OSCC, which makes up over 90% of all oral tumors, may originate in several intraoral locations, such as the tongue, gingiva, lips, buccal mucosa, hard and soft palate, and the floor of the mouth (1).

OSCC persists as a major public health concern, with limited improvement in prognosis over the years and a global five-year survival rate of roughly 50% (2). The unfavorable prognosis is largely explained by delayed detection and the aggressive clinical course of advanced tumors. This situation highlights the pressing need for improved diagnostic methods and the exploration of novel therapeutic strategies (2). Although tobacco use, alcohol intake, and HPV infection are recognized as major risk factors, the influence of genetic predisposition in the initiation and progression of OSCC is gaining increasing research interest (3).

Globally, OSCC remains a major public health concern, particularly in South and Southeast Asia, where high prevalence is linked to tobacco use, betel quid chewing, alcohol consumption, and poor oral hygiene (4, 5). Conventional OSCC in older patients is strongly associated with tobacco and alcohol exposure; however, human papillomavirus (HPV) infection has been linked to oropharyngeal tumors and atypical cases in younger individuals (4).

The poor survival outcomes are largely attributed to late diagnosis and the rapid progression of advanced-stage tumors. This highlights the need for improved early detection tools and novel therapeutic strategies. OSCC typically develops gradually, beginning with potentially malignant disorders (PMDs) such as leukoplakia, oral submucous fibrosis, or oral erythroplakia, which may progress to invasive carcinoma (6). These conditions are increasingly recognized as important factors in oral carcinogenesis (7, 8). To preserve the integrity of the oral epithelium and avoid malignant transformation, vitamin D's biological actions, which include controlling cell proliferation, apoptosis, differentiation, and immune response, are mediated in large part by the VDR (6). Genetic factors influencing these early cellular changes are essential to understand, as they may serve as biomarkers for early detection, risk stratification, and targeted preventive strategies (9).

Vitamin D exerts most of its genomic effects through the VDR, which belongs to the nuclear hormone receptor family (10).Upon binding with its active ligand, 1,25-dihydroxyvitamin D₃ (calcitriol), the VDR forms a heterodimer with the retinoid X receptor (RXR), thereby controlling the transcription of several downstream genes essential for maintaining cellular homeostasis (10).Because of its recognized anti-proliferative, pro-differentiative, anti-inflammatory, and pro-apoptotic properties, VDR signaling is essential for maintaining oral epithelial integrity (6).

Genetic variations in VDR, particularly SNPs such as *FokI* (rs2228570), *TaqI* (rs731236), and *ApaI* (rs7975232), may affect receptor function and modulate susceptibility to OSCC. Variants in genes involved in vitamin D metabolism, including *CYP24A1,* which encodes an enzyme that catabolizes active vitamin D metabolites, may also influence disease risk (11, 12). Understanding the role of these genetic factors is critical for identifying biomarkers for early detection, risk stratification, and potential targeted interventions.

Therefore, this systematic review aims to synthesize existing evidence to better understand how gene polymorphisms involved in the vitamin D pathway, particularly in the VDR and CYP24A1 genes, contribute to the onset and progression of OSCC and potentially malignant disorders. Therefore, this systematic review aims to synthesize existing evidence on the association between VDR and CYP24A1 gene polymorphisms and susceptibility to OSCC and potentially malignant disorders.

## Methods

### Registration

This systematic review was prospectively registered in PROSPERO (CRD420251105619) on 21/07/2025 prior to study initiation, and conducted in accordance with the PRISMA 2020 guidelines (13).

### Publication search strategy

A systematic search was conducted to identify relevant studies investigating the association between VDR gene polymorphisms and OSCC. Databases searched included PubMed, Google Scholar, and Web of Science from 2012 to 2025. Keywords and their combinations included: “VDR”, “SNP”, and “OSCC”. Boolean operators were used where applicable (e.g., “VDR” AND “SNP” AND “OSCC”). Only studies published in English and involving human subjects were considered. Reference lists of selected articles were manually screened to identify additional eligible studies. Duplicate records from multiple databases were removed before screening.

### Study selection

#### Inclusion criteria

The selection of studies was based on the following criteria (1): inclusion of case-control study designs, (2) availability of original data, and (3) publication in the English language. This review incorporated only those studies that satisfied all eligibility requirements.

#### Exclusion criteria

Studies listed below were not included: (1) Review articles, duplicate publications, and abstracts without full text. (2) Studies without reported genotype frequencies. (3) Animal studies or *in vitro*/cell line studies. (4) Cross-sectional studies limited to case reports. (5) Studies that did not perform genetic analysis of VDR polymorphisms.

### Selection process

Two reviewers independently screened all titles, abstracts, and full texts. Disagreements were resolved through discussion and consensus. The methodological quality of included studies was assessed using the NOS. Studies fulfilling 7–9 criteria were considered low risk of bias, those meeting 5–6 criteria were moderate risk, and studies with fewer than 5 criteria were considered high risk.

### Risk-of-bias assessment

**Figure 1:**
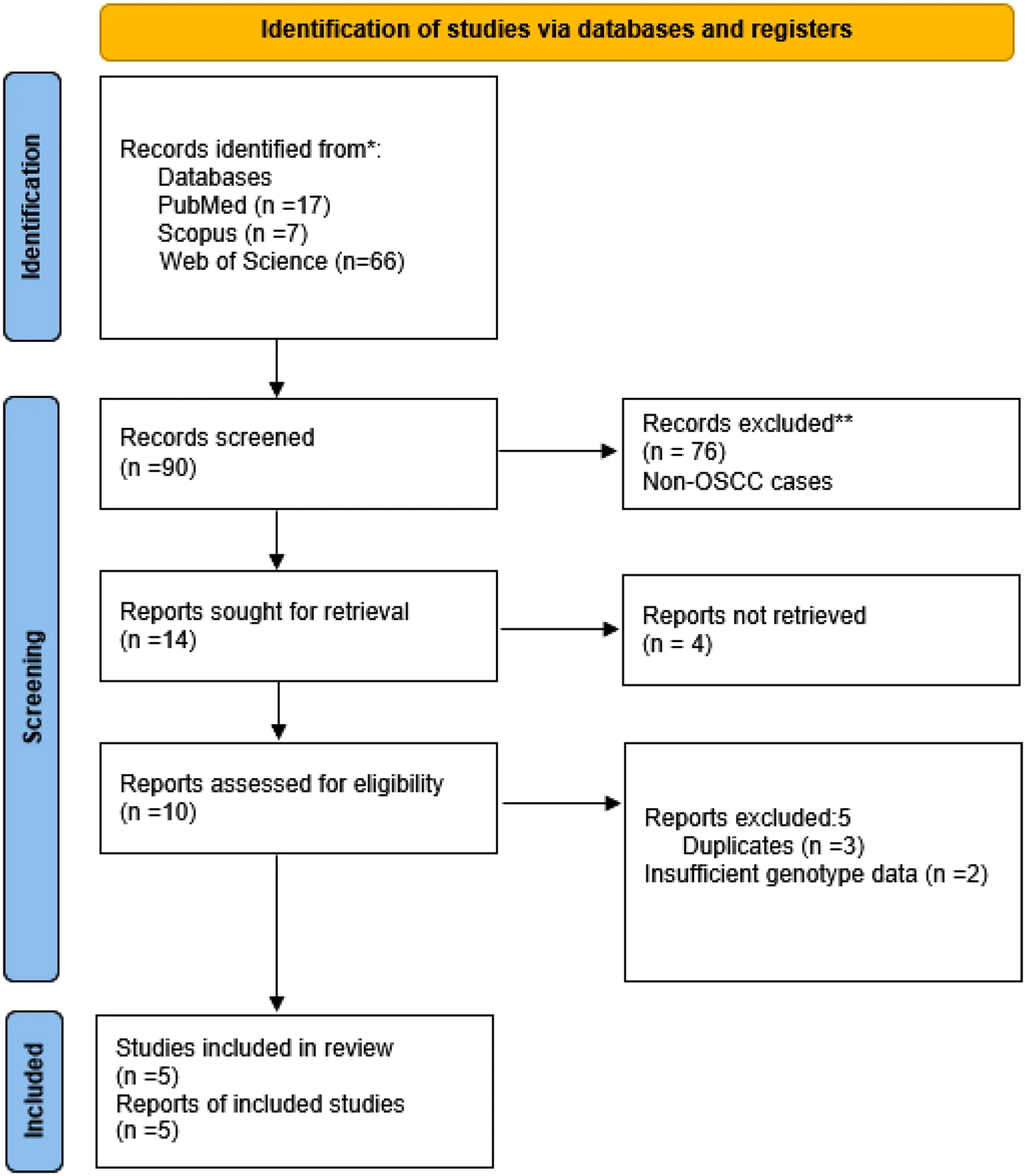
Newcastle–Ottawa scale (NOS) assessment showing the quality of included studies across selection, comparability, outcome, and overall domains. Studies were rated as Good, Fair, or Poor based on NOS criteria.

NOS was used to assess methodological quality. Studies meeting 7–9 criteria were classified as low risk of bias, 5–6 as moderate risk, and fewer than 5 as high risk. A detailed risk-of-bias summary for each study, including its potential impact on the overall findings, is provided in Table 1; Figure 1.

**Table 1:** Results from the Newcastle–Ottawa risk assessment for observational studies.

Study (Author, Year)	Selection	Comparability	Outcome	Total Score (/9)
Zeljic et al. (2012) (12)	★★★★	★	★★	7
Małodobra-Mazur et al. (2012) (20)	★★★	★	★★	6
Shen et al. (2020) (14)	★★★★	★★	★★	8
Nigam et al. (2023) (15)	★★★★	★★	★★	8
Mohtasham et al. (2024) (16)	★★★★	★	★★	7

The star rating represents study quality based on the NOS, with a maximum of 9 stars: 4 stars for Selection, 2 stars for Comparability, and 3 stars for Outcome assessment.

Overall, most included studies demonstrated a low to moderate risk of bias, particularly in the domains of selection and outcome assessment, which supports the internal validity of the reported associations. However, variability in comparability scores reflects incomplete adjustment for key confounding factors such as tobacco use, alcohol consumption, betel quid chewing, sun exposure, and serum vitamin D levels. These limitations may have influenced the magnitude and direction of the observed genetic associations. Therefore, although the findings suggest an association between VDR and CYP24A1 polymorphisms and susceptibility to OSCC, the results should be interpreted with caution.

### Data synthesis

Due to heterogeneity across studies in terms of populations, SNPs examined, and outcome measures, a meta-analysis was not performed. Findings were summarized narratively in accordance with PRISMA 2020 guidelines.

## Results

**Figure 2:**
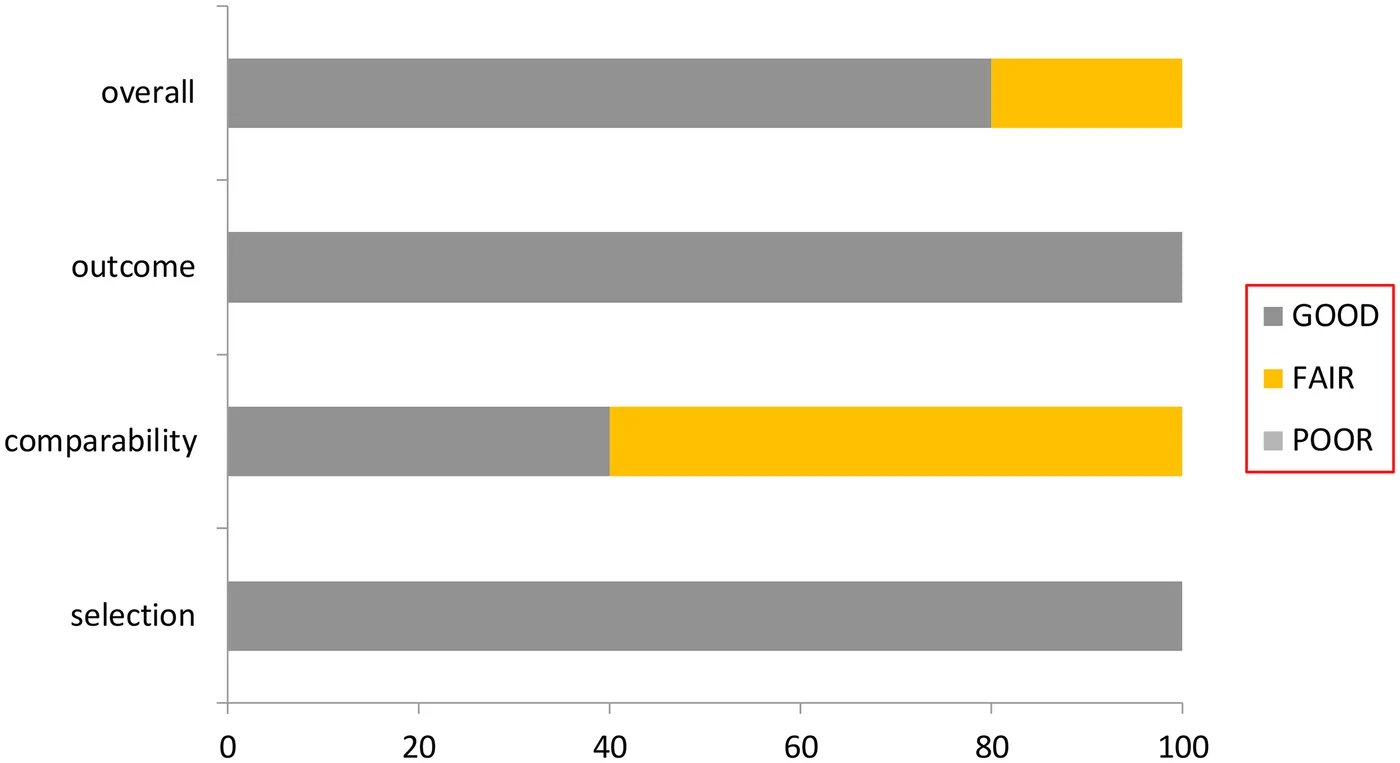
PRISMA 2020 flow diagram showing the identification, screening, eligibility assessment, and inclusion of studies evaluating VDR polymorphisms and OSCC susceptibility. Numbers at each stage represent records identified, screened, excluded, and included in qualitative synthesis.

The systematic database search in PubMed, Scopus, and Web of Science yielded 90 records. During title and abstract screening, 76 records were excluded as they were not associated with OSCC. Fourteen were selected for full-text assessment; however, four could not be retrieved. Of the ten articles reviewed in full, five were excluded: three due to duplication and two for not meeting the defined inclusion criteria. Consequently, five studies fulfilled the eligibility criteria and were incorporated into the qualitative synthesis of this review (Figure 2: PRISMA 2020 flow diagram).

Five papers that met all of the inclusion criteria made up the final synthesis and were thus included. Table 2 summarizes the characteristics of the included studies. The studies were conducted in Serbia, Poland, Iran, North India, and China, with sample sizes ranging from 29 to 207 cases. Key SNPs examined included *FokI* (rs2228570), *TaqI* (rs731236), *ApaI* (rs7975232), and *CYP24A1* (rs2296241). Genotyping methods varied across studies, including PCR-RFLP, TaqMan allelic discrimination, and minisequencing. According to the included studies' quality evaluation, most of them showed a low risk of bias in almost every NOS (Figure 1). The included studies consistently demonstrated that specific VDR and *CYP24A1* polymorphisms are associated with OSCC susceptibility. In particular, the *FokI* (rs2228570) variant was linked to poorer survival outcomes in certain populations, while the *TaqI* (rs731236) CC genotype was associated with higher tumor differentiation in the North Indian cohort. The *ApaI* (rs7975232) polymorphism showed a positive association with OSCC risk in populations from Iran and China, and the *CYP24A1* (rs2296241) heterozygous variant appeared protective in the Serbian cohort. No major contradictory findings were reported, though differences across studies likely reflect population-specific genetic variations, sample sizes, and methodological diversity. Overall, these findings support a role for VDR and *CYP24A1* polymorphisms in modulating OSCC susceptibility, potentially influencing key cellular processes such as proliferation, apoptosis, and immune regulation, and highlighting their utility as biomarkers for risk stratification and early detection.

**Table 2:** Description of individual study.

Author (Year)	Country	Sample Size (Cases/Controls)	SNPs Studied	Genotyping Method	Key Findings
Zeljic et al. (2012) (12)	Serbia	110/122	*FokI*(rs2228570) *CYP24A1*(rs2296241)	PCR-RFLP, RTq PCR	*CYP24A1*(rs2296241) protective; *FokI* ff genotype linked to poor survival
Małodobra-Mazur et al. (2012) (20)	Poland	73/100	rs2238135,rs2107301	Minisequencing + electrophoresis	rs2238135 G/C genotype associated with increased OSCC risk
Shen et al. (2020) (14)	China	177/207	rs731236,rs739837, rs757343,rs2107301,rs2239185,rs7975232,rs11574129 and rs11568820	TaqMan allelic discrimination	Intronic variant rs2239185 TT and *ApaI* (rs7975232) CC genotypes increased OLP risk
Nigam et al. (2023) (15)	India	72 OSCC/300 controls	*TaqI* (rs731236)	PCR-RFLP	*TaqI* CC genotype linked to higher tumor differentiation and protective effect
Mohtasham et al. (2024) (16)	Iran	29 OSCC/40 controls	*ApaI* (rs7975232)	PCR-RFLP	*ApaI*(rs7975232) is positively associated with OSCC susceptibility

Heterogeneity across study populations and the specific SNPs analyzed precluded the possibility of performing a meta-analysis. Additionally, small sample sizes and limited adjustment for lifestyle and environmental factors, such as tobacco and alcohol use, sun exposure, and dietary vitamin D intake, may restrict the generalizability of the findings. Furthermore, the inclusion of only English-language publications introduces the potential for language bias.

## Discussion

The results of this review highlight the complex role of variations in the VDR gene in susceptibility to OSCC. Evidence from five studies across multiple populations indicates that specific VDR polymorphisms, including *FokI, TaqI, ApaI,* and *CYP24A1,* may influence receptor function and gene expression, thereby affecting critical cellular processes such as proliferation, apoptosis, and immune regulation. However, the strength of these conclusions is moderated by study-level bias, particularly incomplete confounder adjustment and small sample sizes, underscoring the need for cautious interpretation of the findings.

Evidence from studies conducted in Serbia, Poland, Iran, North India, and China supports these findings, highlighting population-specific differences in VDR polymorphisms and their potential role in oral carcinogenesis. Variations in the VDR gene have emerged as important genetic determinants influencing susceptibility to OSCC. VDR regulates cell proliferation, apoptosis, immune function, and inflammatory responses, which are critical for oral epithelial homeostasis. Polymorphisms in the VDR gene may alter receptor expression or function, potentially reducing the protective effects of vitamin D. Although VDR genetic variations are associated with OSCC risk, this study did not account for environmental and lifestyle factors such as tobacco use, alcohol consumption, betel quid chewing, poor oral hygiene, sun exposure, and dietary vitamin D intake. Collectively, the five included studies underscore the significant influence of VDR genetic variations on OSCC susceptibility, while highlighting the interplay between genetic predisposition, population-specific factors, and unmeasured environmental and lifestyle influences in oral carcinogenesis.

Evidence from a Serbian cohort demonstrated significant allele frequency differences in VDR, *CYP27B1,* and *CYP24A1* polymorphisms, suggesting their possible contribution to disease risk. Notably, the *CYP24A1* rs2296241 heterozygous variant was associated with a substantially lower incidence of OSCC (OR = 0.281, *P* = 0.00001), with carriers showing nearly a 72% reduction in risk compared to those with the wild-type genotype (12).

In a Polish population (11), examined the relationship between VDR gene variants and susceptibility to OSCC. The study focused on two specific SNPs: rs2238135 and rs2107301. Among patients with OSCC, the G/C heterozygous genotype of rs2238135 was found to be the most prevalent and was significantly associated with a 3.16 times greater risk of disease development (OR = 2.68 and OR = 2.25, respectively). Others have reported similar results (17). The polymorphism rs2107301 did not significantly affect genotype frequency or raise the risk of OSCC. These results highlight the need for more investigation into the functional significance of VDR variations in oral carcinogenesis and support the possible relevance of the *FokI* polymorphism as a susceptibility marker. In this article, we obtained comparable results (11).

The association between OSCC and polymorphisms in the VDR gene in a population from Iran. The study examined the *ApaI* (rs7975232) polymorphism, although the *ApaI* polymorphism exhibits a positive association with susceptibility to OSCC. These results further imply population-specific genetic factors in OSCC development and corroborate earlier findings that connected *ApaI* to OSCC susceptibility (16).

The study by Shen et al. (14) provided evidence linking VDR polymorphisms to the risk of oral lichen planus (OLP), a potentially malignant disorder that can progress to OSCC. The rs2239185 TT and *ApaI* (rs7975232) CC genotypes were significantly associated with an increased OLP risk, with individuals carrying the CC haplotype (rs2239185, rs7975232) showing even higher susceptibility (OR = 3.11, *P* = 0.005). These findings highlight the cumulative effect of specific VDR SNP combinations and underscore the importance of gene–gene interactions in the early stages of oral carcinogenesis and progression to OSCC.

The *TaqI* polymorphism (rs731236, T > C) of the VDR gene has been associated with differences in susceptibility to OSCC and premalignant oral diseases in the North Indian cohort. Evidence suggests that the C allele may lower the risk of developing such illnesses, notably leukoplakia, but the CC genotype may be associated with higher tumor grades at presentation. Furthermore, this SNP exemplifies a gene-environment interaction because its impact on illness risk appears to be altered by smoking habits (15).

Mohtasham et al. (16) showed substantial age-related differences between controls and OSCC patients (*P* = 0.001), as well as a strong correlation between the rs7975232 Aa and aa genotypes and OSCC risk (OR = 17.33 and OR = 8.67, respectively). These findings provide more evidence for the role of genetic predispositions in mouth cancer risk. These studies highlight the role of genetic polymorphisms in regulating susceptibility to oral illnesses, with some variants offering protection and others increasing risk.

Despite these consistent findings, variability exists, particularly for *TaqI, ApaI,* and *BsmI,* likely due to differences in study design, sample size, genotyping methods, and population diversity. Small cohorts and unreported lifestyle factors limit generalizability (18, 19). Heterogeneity and methodological differences precluded meta-analysis, and inclusion of only English-language publications may have introduced language bias.

This systematic review has certain limitations that should be noted. The small number of included studies limits the conclusions' generalizability. The lack of a meta-analysis further reduces the quantitative strength of the evidence. Furthermore, the ethnic diversity of the cohorts studied makes it difficult to identify population-specific genetic effects and potential gene-environment interactions.

Important confounding factors such as serum vitamin D levels, dietary habits, sunshine exposure, tobacco use, and alcohol consumption, all of which might alter the vitamin D pathway, were not systematically addressed across studies. Furthermore, methodological variability, such as differences in genotyping procedures, sample sizes, and the specific SNP panels studied, was not thoroughly addressed for the potential to create bias. Future research should prioritize well-powered, multiethnic studies with standardized procedures that account for environmental and lifestyle variables to determine the true impact of VDR and related gene variants on OSCC susceptibility.

In conclusion, the five included studies collectively support the relevance of VDR polymorphisms in OSCC and OPMD susceptibility, with effects modulated by environmental and lifestyle factors. While certain SNPs confer increased risk or protection in specific populations, heterogeneity and methodological limitations underline the need for larger, integrated studies to validate these findings and explore their potential clinical applications.

## Conclusion

This systematic review underscores the possible involvement of VDR gene variants, particularly *FokI, TaqI, ApaI, and CYP24A1*, in influencing OSCC susceptibility. Evidence from five high and moderate-quality case-control studies suggests that these SNPs may influence individual susceptibility and, in some cases, prognosis. The observed associations underscore the importance of host genetic factors in oral carcinogenesis, likely through disruption of vitamin D-mediated pathways related to immune response, cell proliferation, and apoptosis.

Despite promising findings, limitations, including small sample sizes, geographic heterogeneity, and limited adjustment for confounders, caution against definitive conclusions. Future research should prioritize well-powered, multi-ethnic studies with strong control over lifestyle variables, including smoking habits and vitamin D levels.

Identifying VDR gene variants associated with OSCC risk may eventually support risk stratification, early detection, particularly in populations at risk, and personalized prevention strategies as well.

## Data Availability

The datasets presented in this study can be found in online repositories. The names of the repository/repositories and accession number(s) can be found in the article/Supplementary Material.
